# *Panax ginseng* C.A. Meyer (Rg3) Ameliorates Gastric Precancerous Lesions in Atp4a^−/−^ Mice via Inhibition of Glycolysis through PI3K/AKT/miRNA‐21 Pathway

**DOI:** 10.1155/2020/2672648

**Published:** 2020-01-31

**Authors:** Wei Liu, Hua-feng Pan, Liang-jun Yang, Zi-ming Zhao, Dong-sheng Yuan, Yuan-liang Liu, Li-zhu Lin

**Affiliations:** ^1^Guangzhou University of Chinese Medicine, Guangzhou 510405, China; ^2^First Affiliated Hospital, Guangzhou University of Chinese Medicine, Guangzhou 510095, China; ^3^Guangdong Province Engineering Technology Research Institute of T.C.M., Guangzhou 510095, China

## Abstract

Gastric cancer, one of the most common types of cancers, develops over a series of consecutive histopathological stages. As such, the analysis and research of the gastric precancerous lesions (GPLs) play an important role in preventing the occurrence of gastric cancer. Ginsenoside Rg3 (Rg3), an herbal medicine, plays an important role in the prevention and treatment of various cancers. Studies have demonstrated a correlation between glycolysis and gastric cancer progression. Herein, the aim of the present study was to clarify the potential role for glycolysis pathogenesis in Rg3-treated GPL in Atp4a^−/−^ mice. The GPL mice model showed chronic gastritis, intestinal metaplasia, and more atypical hyperplasia in gastric mucosa. According to the results of HE and AB-PAS staining, it could be confirmed that GPL mice were obviously reversed by Rg3. Additionally, the increased protein levels of PI3K, AKT, mTOR, HIF-1*α*, LDHA, and HK-II, which are crucial factors for evaluating GPL in the aspect of glycolysis pathogenesis in the model group, were downregulated by Rg3. Meanwhile, the miRNA-21 expression was decreased and upregulated by Rg3. Furthermore, the increased gene levels of Bcl-2 and caspase-3 were attenuated in Rg3-treated GPL mice. In conclusion, the findings of this study imply that abnormal glycolysis in GPL mice was relieved by Rg3 via regulation of the expressions of PI3K, AKT, mTOR, HIF-1*α*, LDHA, HK-II, and miRNA-21. Rg3 is an effective supplement for GPL treatment and can be harnessed to inhibit proliferation and induce apoptosis of GPL cells.

## 1. Introduction

Globally, gastric cancer is a serious healthcare problem because of its high mortality rate [1, 2]. The prevalence in China is high, with 42% of all worldwide cases occurring in China [3]. Gastric precancerous lesions (GPLs) are a premalignant stage within Correa's cancer cascade and are recognized as a point of no return for gastric carcinogenesis [4]. Studies have shown a significantly increased risk of gastric cancer in patients with either gastric atrophy (5.8 times the risk of gastric cancer compared to individuals without gastric atrophy) [5] or GPL (10 times the risk of gastric cancer compared to individuals with no evidence of GPL) [6]. Although various therapies exist for the treatment of GPL and gastric cancer, the therapies are less than satisfactory. This may be due to the multiple genetic variations and multiple altered microenvironments (such as altered glucose metabolism) that can promote gastric carcinogenesis.

Reprogrammed energy metabolism is a cancer hallmark [7]. As such, dysfunctional changes in cellular energy metabolism in GPL are worthy of investigation. Unlike normal cells, cancer cells preferentially metabolize glucose to lactate through glycolysis even in the presence of oxygen, the phenomenon known as the Warburg effect [8]. Emerging evidence has shown that metabolic alterations are critical to the growth, proliferation, and survival of tumor cells, suggesting alterations in GPL glycolysis. Studies have shown that pathways such as PI3K/AKT/mTOR and HIF-1*α* are central regulators of glycolysis, cancer metabolism, and cancer cell proliferation [9]. Also, of note, miRNA-21, a circulating tumor biomarker for early cancer diagnosis, is capable of mediating the expression of Bcl-2 and PI3K/AKT/mTOR signaling pathway [10–13]. In this study, Atp4a^−/−^ mice were chosen to provide a model of GPL, based on a previous report [14] to investigate whether glycolysis occurs in GPL.

Ginseng, the root of *Panax ginseng* C.A. Meyer has been widely used in East Asian countries for thousands of years as a natural tonic [15]. Ginsenoside Rg3 (Rg3), the main active component of ginseng, is a four-ring steroid-like molecule with attached sugar moieties. Extracts of *Panax ginseng* C.A. Meyer have been shown to have significant physiological effects [16] such as hepatoprotection, neuroprotection, cardiovascular protection, and the promotion of immunity, as well as antifatigue, antioxidant, and, most importantly, antitumor effects [17–20]. These extracts induce apoptosis and decrease the growth of tumor cells, inhibiting the invasion and metastasis of various cancers including gastric, intestinal, and lung cancers [21]. The gastric mucosal protective effect of Rg3 on GPL has not been demonstrated.

Glycolysis is recognized as an essential energy source for cancer progression. Thus, we hypothesize that abnormal glycolysis precedes GPL. To assess this possibility, we investigated the levels of proteins involved in the PI3K/AKT/mTOR pathway, which were crucial factors for evaluating GPL in the aspect of glycolysis pathogenesis. Importantly, we investigated the therapeutic role of Rg3 in GPL treatment via inhibiting the glycolysis process through PI3K/AKT/mTOR pathway downregulation and miRNA-21 targeting and analyzed the effects of Rg3 to induce cell apoptosis in Atp4a^−/−^ mice treated for gastric cancer cells.

## 2. Materials and Methods

### 2.1. Mice and Treatment

Thirty male Atp4a^−/−^ C57Bl/6 mice (8-week-old, weighing 20–25 g) were generated using CRISPR/Cas9 (Shanghai Model Organisms Center, Inc.).

CRISPRs were designed using a CRISPR design web tool (http://crispr.mit.edu). The CRISPR process involved a single-guide RNA (sgRNA) sequence targeting Atp4a and the primers gHKA-5′ (ACAGCAGAAAGTATCTGTTGTTG), gHKA-3′ (GCATAAAGGAGGGTAATGGTAG), and NEO (5′-TCCAGAATGTCCTCAATCTACT). All of the mice were provided with food and water under specific-pathogen-free conditions at approximately 24 ± 1°C with 40–80% relative humidity. All experiments were carried out in accordance with guidelines of the Guangzhou University of Chinese Medicine. The study protocol was approved by the Ethical Committee on Animal Research at Guangzhou University of Chinese Medicine (ref. S2017089). All efforts were made to minimize the suffering of animals as much as possible.

Rg3 (purity ≥ 98%) was obtained from Jilin Yatai Pharmaceutical Co., Ltd. (Jilin, China). After 2 weeks of adaptation to the conditions, histological assessments were carried out at 10 weeks. Subsequently, 10-week-old Atp4a^−/−^ mice and wild-type (WT) mice from the same litter were divided into four groups as follows:

1. Control group (*n* = 10): each WT mouse received intragastric administration of an equivalent volume of distilled water.
2. Model group (*n* = 10): each Atp4a^−/−^ mouse was given an equivalent volume of distilled water, respectively, by gavage daily.
3. High-dose Rg3 group (*n* = 10): each Atp4a^−/−^ mouse received intragastric administration of Rg3 (10 mg/kg) daily for 10 weeks.
4. Low-dose Rg3 group (*n* = 10): each Atp4a^−/−^ mouse received intragastric administration of Rg3 (5 mg/kg) daily for 10 weeks.

**Figure 1:**
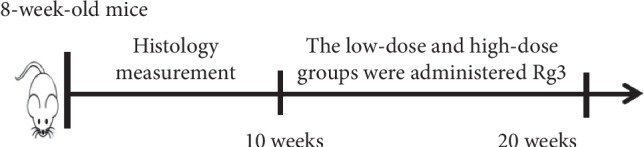
Experimental design (*n* = 10 mice per group).

After 10 weeks of treatment, the mice were sacrificed by inhalation of CO_2_ (Figure 1).

### 2.2. Primary Antibodies

The following primary antibodies were obtained to assess the expression of proteins related to the phosphoinositide 3-kinase (PI3K)/AKT/mechanistic target of rapamycin (mTOR) signaling pathway and the glycometabolic-related proteins hypoxia-inducible factor- (HIF-) 1*α*, hexokinase- (HK-) II, and lactate dehydrogenase A (LDHA): P-PI3K (Abcam ab86714), p-AKT (Cell Signaling Technology 4067), P-mTOR (Cell Signaling Technology 5536), HIF-1*α* (Millipore MAB538), LDHA (Abcam ab101562), HK-II (Abcam ab209847), and *β*-actin (Abcam ab8227).

### 2.3. Histological Evaluation

After 10 weeks of treatment, gastric tissues were dissected from selected mice (*n* = 3 mice per group), fixed in 10% formalin for 2 days, dehydrated using a serial alcohol gradient, embedded in paraffin, cut into 4 *μ*m sections, and stained with either hematoxylin and eosin (HE) or periodic acid–Schiff (PAS) and Alcian blue (AB) for light microscopy. Four types of GPL-related histological features were noted: chronic superficial gastritis, chronic atrophic gastritis, intestinal metaplasia, and tumor.

### 2.4. Western Blot Analysis

Gastric mucosal tissues (*n* = 6 mice per group) were homogenized and lysed in the sample buffer (0.5 M Tris/HCl, pH 6.8, 50% glycerol, 10% sodium dodecyl sulfate (SDS), and an inhibitor protease and phosphatase cocktail at a ratio of 1 : 100). The lysate was then centrifuged at 12,000 rpm for 10 min at 4°C and then denatured by boiling at 100°C with the loading buffer at a ratio of 1 : 4. Equal amounts of protein from each sample (40 *μ*g) were then separated by 10% SDS-polyacrylamide gel electrophoresis (PAGE) and transferred to polyvinylidene fluoride membranes. The membranes were then blocked with 5% bovine serum albumin (BSA) in Tris-buffered saline with Tween 20 (TBST) for 1 h at room temperature. The membranes were incubated overnight at 4°C with primary antibodies. Horseradish peroxidase-conjugated goat anti-rabbit secondary antibodies were then added for 1 h at room temperature. The protein expression was assessed using a superenhanced chemiluminescence (ECL) reagent (K003, Affinity Biosciences, Cincinnati, OH, USA). *β*-Actin was used as the internal control. Acquired images were analyzed using Image Lab software (version 3.0, Bio-Rad Laboratories, Inc., USA).

### 2.5. Quantitative Real-Time Polymerase Chain Reaction (qRT-PCR)

To evaluate the effects of Rg3 on the apoptosis-related gene expression, the mRNA expression of Bax, Bcl-2, and caspase-3 and microRNA-21 (miRNA-21) expression were examined. Total RNA was extracted from frozen stomach samples (*n* = 3 mice per group) using the Trizol reagent in accordance with the manufacturer's specifications. RNA (1.0 *μ*g) was reverse transcribed into complementary DNA (cDNA) using the iQ5 Multicolor Real-Time PCR Detection System (Bio-Rad, USA).The cDNA was amplified using specific primers (Table 1) for 40 cycles under the following conditions: 95°C for 300 s, annealing at 95°C for 15 s, and 60°C for 32 s, with the final incubation at 60°C for 7 min. Bax, Bcl-2, and caspase-3 mRNA expression levels were calculated using the 2^−ΔΔCt^ method and normalized to 18s RNA levels. miRNA-21 expression levels were calculated using the 2^−ΔΔCt^ method and normalized to U6B snRNA levels.

### 2.6. Statistical Analysis

The results are presented as mean ± standard error of the mean (SEM). Two-way ANOVA followed by Dunnett's post hoc test was used for the analysis of differences between groups. The S-N-K test was used when the variance was uniform, and the rank-sum test was used when the variance was not uniform. *P* value < 0.05 was defined as statistically significant. Data were analyzed using SPSS 20.0 software.

## 3. Results

### 3.1. Histopathological Changes of the Gastric Mucosa

**Figure 2:**
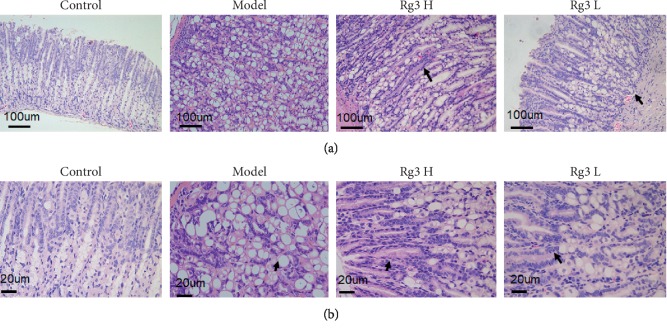
Histopathological changes of the gastric mucosa in various groups. HE staining: (a) ×200; (b) ×400.

Histological observation utilizing HE was used to evaluate gastric mucosal lesions. Compared to the WT control group, the gastric mucosa of the Atp4a^−/−^ mice was not complete, elasticity of the gastric wall was poor, and there were differences in basement membrane thickness. Within the disorganized gastric mucosal tissue, enlarged and dilated glands were found. Furthermore, the size of the gastric mucosal epithelial cells varied, and they had obvious morphological heterogeneity. Mesenchymal tissues were infiltrated by inflammatory cells. The number of dysplastic glands was significantly increased, and they were irregularly arranged and weakly stained. Taken together, these results demonstrate diffuse gastric epithelial dysplasia in the model group. No ulcers or papillomas were observed, but the mesenteric vasculature was quite prominent. Importantly, in both the high- and low-dose Rg3 groups, dysplasia of the gastric epithelial cells was less pronounced, had a scattered distribution, and was confined to the basement membrane side. These results suggest that Rg3 protected the gastric mucosa of Atp4a^−/−^ mice (Figure 2).

**Figure 3:**
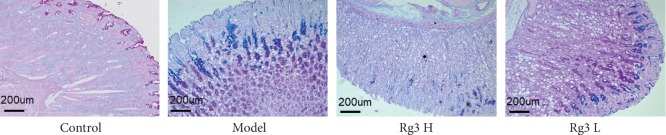
Histopathological changes of the gastric mucosa in various groups. AB-PAS staining: ×100.

AB-PAS staining was used to evaluate the types of intestinal metaplasia. In the model group, mucosal metaplastic tissue was positive for AB-PAS staining, indicating replacement of fundic parietal and chief cells by foamy cells containing neutral and acid mucins (Figure 3). The gastric mucosal metaplastic tissue had a wide range of lesions and deep staining, with the basal side showing mainly intestinal metaplasia. Within the gastric cavity, intestinal metaplasia was of incomplete type, while within the lamina propria, intestinal metaplasia was of complete type. Importantly, after treatment with Rg3, there was a significant reduction in GPL compared to that in the model mice. This was particularly the case for crowded tubular glandular structures and back-to-back tubular structures, which represent the intermediate phase between GPL and gastric cancer.

### 3.2. Effects of Rg3 on Glycometabolic-Related Protein Expression in Atp4a^−/−^ Mice

**Figure 4:**
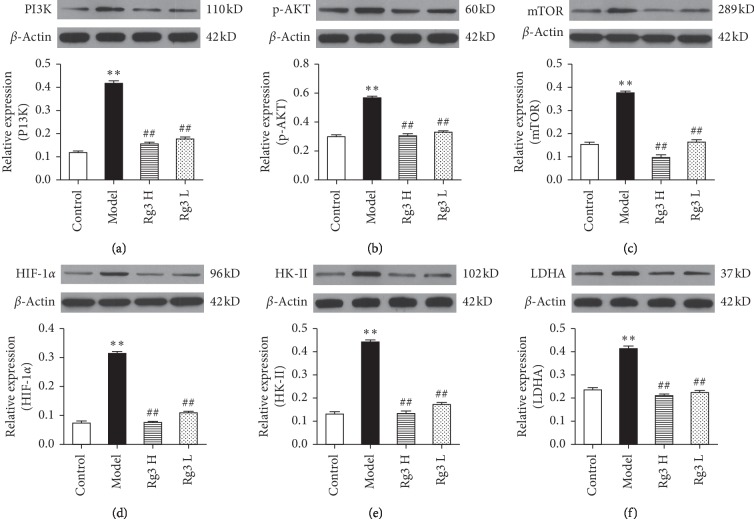
Protein expression of glycometabolic-related proteins in the gastric mucosa of Atp4a^−/−^ mice treated with Rg3 based on western blotting results and quantification of band intensities in each blot for (a) PI3K, (b) p-AKT, (c) mTOR, (d) HIF-1*α*, (e) HK-II, and (f) LDHA. Upregulation of PI3K, p-AKT, mTOR, HIF-1*α*, HK-II, and LDHA proteins in the gastric mucosa of Atp4a^−/−^ mice compared to levels in the WT control group. Data are shown as mean ± SEM (*n* = 6). ^*∗∗*^*P* < 0.01 versus the WT control group. ^##^*P* < 0.01 versus the model group.

Research has shown that Atp4a^−/−^ parietal cells store large amounts of glycogen. Hence, glycometabolic-related proteins associated with the Warburg effect were assessed by western blot analysis, along with PI3K/AKT signaling pathway-related proteins.  Figure 4 shows that the protein levels of PI3K, p-AKT, mTOR, HIF-1*α*, HK-II, and LDHA were significantly increased in the model group compared to the WT control group. At both low and high doses, the 10-week Rg3 treatment significantly decreased levels of PI3K, p-AKT, mTOR, HIF-1*α*, HK-II, and LDHA compared to the levels in the model group.

### 3.3. Effects of Rg3 on Apoptosis-Related Gene Expression in Atp4a^−/−^ Mice

**Figure 5:**
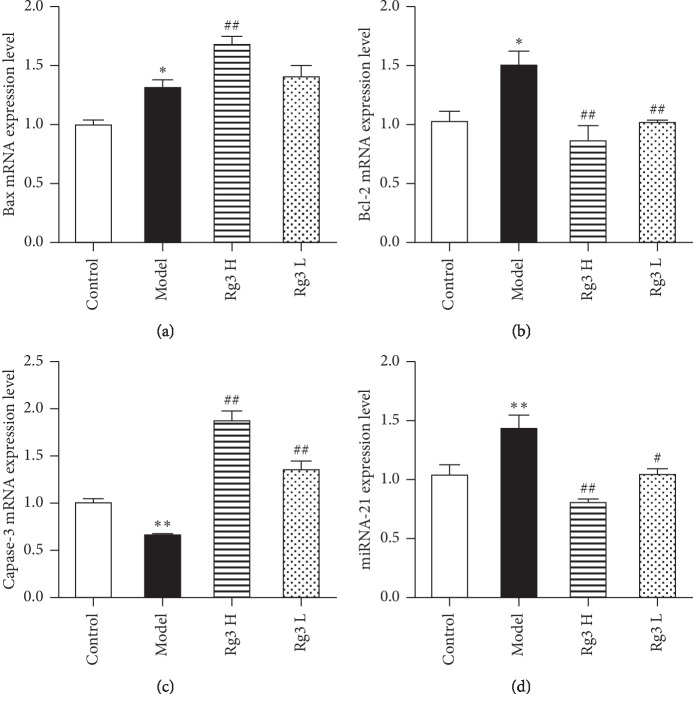
Effects of Rg3 on the apoptosis-related gene expression in Atp4a^−/−^ mice. qRT-PCR results for (a) Bax, (b) Bcl-2, (c) caspase-3, and (d) miRNA-21 expressions in Atp4a^−/−^ mice after treatment with Rg3. Data are shown as mean ± SEM (*n* = 4 mice per group). ^*∗*^*P* < 0.05 and ^*∗∗*^*P* < 0.01 versus the WT control group. ^#^*P* < 0.05 and ^##^*P* < 0.01 versus the model group.

To evaluate the effects of Rg3 on the apoptosis-related gene expression, the mRNA expression of Bax, Bcl-2, and caspase-3 and the miRNA-21 expression were examined. Bax, Bcl-2, and miRNA-21 expressions were upregulated in the Atp4a^−/−^ group compared to the WT control group (*P* < 0.05, *P* < 0.05, and *P* < 0.01, respectively). As shown in Figures 5(a) and 5(b), the Bax expression was markedly increased by high dose (*P* < 0.01), while both doses of Rg3 markedly decreased Bcl-2 (*P* < 0.01) compared to the expression in the model group. As shown in Figure 5(c), the caspase-3 expression in the model group significantly decreased compared to the expression in the WT control group (*P* < 0.01). Rg3 treatment for 10 weeks at both dosages markedly increased the caspase-3 expression when compared to the expression in the model group (*P* < 0.01 for both doses). As shown in Figure 5(d), the miRNA-21 expression was markedly decreased by high-dose (*P* < 0.01) and low-dose (*P* < 0.05) Rg3 compared to the expression in the model group.

## 4. Discussion

*Panax ginseng* C.A. Meyer has been extensively used in Chinese medicine for the treatment of gastric diseases, especially gastric cancer and GPL. Rg3 is an effective chemical trace component of ginseng with a C_42_H_72_O_13_ chemical formula, a molecular weight of 784 Da, and pleiotropic capabilities that include antitumor effects. Previous studies have shown that Rg3 inhibits tumor cell growth, invasion, metastasis, and neovascularization [22–24]. Glycolytic regulation by Rg3, a potential anticancer mechanism, has been previously reported [25, 26]. Pharmacological studies also showed that Rg3 suppressed gastric cancer cell growth [27–29]. Therefore, the effect of Rg3 on glycolysis in GPL tissues was evaluated. In the current study, we found that Rg3 suppressed GPL in the gastric mucosa of Atp4a^−/−^ mice through the regulation of glycolysis. Furthermore, the potential therapeutic mechanism of Rg3 may be associated with its regulation in the PI3K/AKT/miRNA-21 signaling pathway, as well as induction of apoptosis in GPL tissues via the miRNA-21/Bcl-2 pathway.

Although glycolysis is much less efficient at ATP generation than mitochondrial oxidative phosphorylation, glycolysis can provide cancer cells with cellular intermediates for multiple biosynthetic pathways [30, 31]. Hence, targeting glycolysis has emerged as a promising therapeutic strategy for cancer treatment [32]. By identifying and targeting key links in altered glucose metabolism, specific and even individualized therapeutic strategies for gastric cancer may be developed. The PI3K/AKT/mTOR signaling pathway is an essential component of the glycolysis in tumor cells [9]; it upregulates HIF-1*α* and then increases the LDHA expression, thus shifting metabolism to aerobic glycolysis [33]. Recent research has shown the PI3K/AKT/mTOR pathway to be activated in gastric cancer and that activation of this pathway correlated with metastasis, poor prognosis, and reduced survival of gastric cancer patients [34]. The AKT expression directly increases surface translocation of glucose transporters and enhances aerobic glycolysis by prompting HK-II binding to voltage-dependent anion channels in the outer mitochondrial membrane [35]. Upregulation of the PI3K/AKT/mTOR pathway and increased glucose consumption via glycolysis offer advantages to cancer cells during normoxia as well as hypoxia. We found that, in parallel with the PI3K expression, AKT phosphorylation and mTOR were elevated in the Atp4a^−/−^ mice (Figure 4), indicating that PI3K is rapidly induced to activate downstream molecules in the gastric mucosa of GPL. It is consistent with the report that the activation of the PI3K/AKT/mTOR pathway is involved in the procession of GPL [36]. Taken together, Rg3 could reduce the PI3K, AKT, and mTOR protein levels in Atp4a^−/−^ mice, suggesting that the antiangiogenic effect of Rg3 involved the suppression of the PI3K/AKT/mTOR pathway.

HIF-1*α* is downstream of mTOR, which is regulated by PI3K/AKT signaling [10]. HIF-1*α* is a transcriptional activator that acts as a key regulator of the glycolysis and plays an important role in hypoxic responses, inducing the transcription of various genes responsible for tumor angiogenesis, invasion, and metastasis. Overexpressed HIF-1*α* accelerates malignant behaviors in gastric cancer including angiogenesis, invasion, metastasis, and apoptosis [37]. In this study, compared to the HIF-1*α* protein level in WT control mice, elevated HIF-1*α* protein was observed in the model group, which was reduced by Rg3. These data suggest that Rg3 may regulate hypoxic responses in GPL tissues. Taken together, our data suggest that a hypoxic-like environment and organelle homeostasis dysfunction are induced in the precancerous gastric mucosa.

HK-II is the first rate-limiting enzyme in the cellular aerobic glycolysis (AEG) process. The expression of HK-II can reflect the AEG level of cells. High expression of HK-II in malignant tumor tissue maintains high AEG activity, enhancing cell proliferation and invasion [38], and HK-II plays a key role in the activation of the glycolysis. It is acknowledged that intracellular accumulation of lactate results in tumor cell survival and growth [39] and reduced glycolysis [40]. In an anoxic state, LDHA can catalyze the final reversible AEG reaction, which involves converting pyruvate in tumor cells to lactic acid, thus increasing the AEG activity of tumor cells [41]. In addition, lactic acid accumulation leads to microenvironment acidification, which is conducive to tumor survival and growth. Therefore, the LDHA content can be used as a marker of anoxia, neovascularization, and poor tumor prognosis. In our study, both doses of Rg3 significantly reduced HK-II and LDHA protein levels in Atp4a^−/−^ mice. These results indicate that Rg3 may regulate enzymes involved in the cellular glycolysis process in GPL tissues.

The apoptotic cascade increases the risk of gastric cancer [42]. Specifically, the intrinsic apoptotic pathway is related to gastric cancer progression. Therefore, it is reasonable to assume that apoptotic molecules may play a role in the early stages of gastric cancer, including GPL. The intrinsic apoptotic pathway can be activated by various intracellular stimuli, and it relies on the formation of an apoptosome and the activation of Bcl-2 family members, such as Bax and Bcl-2 [43]. In this study, based on qRT-PCR results, the Bax expression was increased and caspase-3 expression was decreased in Atp4a^−/−^ mice compared to the WT control mice. As Rg3 increased the caspase-3 expression, it may mean that Rg3 inhibits cell proliferation and induces apoptosis in GPL tissues.

miRNA-21, which has a high expression in many solid tumors [44], including gastric cancer [10, 45], inhibits the expression of phosphatases, which limits AKT and MAPK signaling. miRNA-21 has also been reported to be a circulating tumor biomarker for early cancer diagnosis [44]. miRNA-21 is involved in cell proliferation, differentiation, and apoptosis, and it is closely related to tumor growth, invasion, and metastasis [46]. miRNA-21 inhibits phosphatase and tensin homolog (PTEN), thereby inhibiting the PI3K/AKT pathway and leading to increased proliferation, cancer cell survival [11], and Bcl-2 expression [13]. In this study, miRNA-21 levels were increased in the model group, which is the first evidence that miRNA-21 is involved in the process of GPL formation. Rg3 downregulated miRNA-21 levels, suggesting that Rg3 may suppress GPL via regulation of the PI3K/AKT/miRNA‐21 pathway. The limitations of this study include the small number of animals that were used. Additionally, off-target effects in Atp4a^−/−^ mice may have influenced the results.

## 5. Conclusions

This is the first study to demonstrate that Rg3 suppresses GPL in Atp4a^−/−^ mice. Altered glucose metabolism is a well-known hallmark of gastric cancer. Based on the data herein, Rg3 inhibits glycolysis through the PI3K/AKT/miRNA‐21 pathway. This study provides new insights into possible metabolic targets that promote gastric cancer and provides data on potential therapeutic strategies for the prevention of gastric cancer.

## Figures and Tables

**Table 1:** Primers used for qRT-PCR.

Gene	Forward primer	Reverse primer	Product size (bp)
Bax	GAACTGGACAGCAATATGGA	GAAGTTGCCATCAGCAAAC	111
Bcl-2	AGCCTTGGCCAGGGAATTAT	GGACTTGGTGCATGGAACAC	160
Caspase-3	GTTCATCCAGTCCCTTTGC	TGTTAACGCGAGTGAGAATG	80
18s	CCTGGATACCGCAGCTAGGA	GCGGCGCAATACGAATGCCCC	112
miRNA-21	ACACTCCAGCTGGGTAGCTTATCAGACTGATG	CTCAACTGGTGTCGTGGA	72
U6B	CTCGCTTCGGCAGCACA	AACGCTTCACGAATTTGCGT	94

## Data Availability

All data generated or analyzed during this study are included in this published article.

## Abbreviations

GPL: Gastric precancerous lesion
HIF-1*α*: Hypoxia-inducible factor-1*α*
Rg3: Ginsenoside Rg3
qRT-PCR: Quantitative real-time polymerase chain reaction
cDNA: Complementary deoxyribonucleic acid.
